# Stroke Telemedicine for Arizona Rural Residents, the Legacy Telestroke Study

**DOI:** 10.1089/tmr.2022.0002

**Published:** 2022-03-14

**Authors:** Bart M. Demaerschalk, Maria I. Aguilar, Timothy J. Ingall, David W. Dodick, Bert B. Vargas, Dwight D. Channer, Erica L. Boyd, Terri E.J. Kiernan, Dennis G. Fitz-Patrick, J. Gregory Collins, Joseph G. Hentz, Brie N. Noble, Qing Wu, Karina Brazdys, Bentley J. Bobrow

**Affiliations:** ^1^ Department of Neurology, Mayo Clinic College of Medicine and Science, Phoenix, Arizona, USA.; ^2^ Center for Connected Care, Mayo Clinic and Center for Digital Health, Mayo Clinic, Rochester, Minnesota, USA.; ^3^ Neuro Hospitalist and Stroke Program, Penrose/St Francis, Centura Health, Colroado Springs, Colorado, USA.; ^4^ Department of Neurology and Neurotherapeutics at University of Texas Southwestern, Dallas, Texas, USA.; ^5^ Department of Research, Mayo Clinic, Phoenix, Arizona, USA.; ^6^ Comprehensive Stroke Program, St. Anthony's Hospital, Lakewood, Colorado, USA.; ^7^ Department of Biostatistics, Mayo Clinic, Phoenix, Arizona, USA.; ^8^ Department of Pharmacy Practice, Oregon State University College of Pharmacy, Corvallis, Oregon, USA.; ^9^ School of Public Health and Nevada Institute of Personalized Medicine at the University of Nevada, Las Vegas, Nevada, USA.; ^10^ Clinical Research Practice, Los Angeles, California, USA.; ^11^ Department of Emergency Medicine, McGovern Medical School at UTHealth, Houston, Texas, USA.

**Keywords:** telestroke, telemedicine, digital health, stroke, thrombolysis, alteplase

## Abstract

**Background:**

Efficacy of telemedicine for stroke was first established by the Stroke Team Remote Evaluation Using a Digital Observation Camera (STRokE DOC) trials in California and Arizona. Following these randomized controlled trials, the Stroke Telemedicine for Arizona Rural Residents (STARR) network was the first telestroke network to be established in Arizona. It consisted of a 7 spoke 1 hub telestroke system, and it was designed to serve rural, remote, or neurologically underserved communities.

**Objective:**

The objective of STARR was to establish a multicenter state-wide telestroke research network to determine the feasibility of prospective collection, recording, and regularly analysis of telestroke patient consultations and care data for the purposes of establishing quality measures, improvement, and benchmarking against other national and international telestroke programs.

**Methods:**

The STARR trial was open to enrollment for 29 months from 2008 to 2011. Mayo Clinic Hospital, Phoenix, Arizona served as the hub primary stroke center and its vascular neurologists provided emergency telestroke consultations to seven participating rural, remote, or underserved spoke community hospitals in Arizona. Eligibility criteria for activation of a telestroke alert and study enrollment were established. Consecutive patients exhibiting symptoms and signs of acute stroke within a 12 h window were enrolled, assessed, and treated by telemedicine. The state government sponsor, Arizona Department of Health Services' research grant covered the cost of acquisition, maintenance, and service of the selected telemedicine equipment as well as the professional telestroke services provided. The study deployed multiple telemedicine video cart systems, picture archive and communications systems software, and call management solutions. The STARR protocol was reviewed and approved by Mayo Clinic IRB, which served as the central IRB of record for all the participating hospitals, and the trial was registered at ClinicalTrials.gov.

**Results:**

The telestroke hotline was activated 537 times, and ultimately 443 subjects met criteria and consented to participate. The STARR successfully established a multicenter state-wide telestroke research network. The STARR developed a feasible and pragmatic approach to the prospective collection, storage, and analysis of telestroke patient consultations and care data for the purposes of establishing quality measures and tracking improvement. STARR benchmarked well against other national and international telestroke programs. STARR helped set the foundation for multiple regional and state telestroke networks and ultimately evolved into a national telestroke network.

**Conclusions:**

Multiple small and rurally located community hospitals and health systems can successfully collaborate with a more centrally located larger hospital center through telemedicine technologies to develop a coordinated approach to the assessment, diagnosis, and emergency treatment of patients manifesting symptoms and signs of an acute stroke syndrome. This model may serve well the needs of patients presenting with other time-sensitive medical emergencies.

Clinical Trial Registration number: NCT00829361.

## Introduction and Background

Efficacy of telemedicine for stroke was first established by the sequentially conducted Stroke Team Remote Evaluation Using a Digital Observation Camera (STRokE DOC) trials in California and Arizona.^1–3^ Following these randomized controlled trials, the Stroke Telemedicine for Arizona Rural Residents (STARR) network was the first telestroke network to be established in Arizona.^4,5^ It was supported by government funding from the Arizona Department of Health Services (ADHS) and originally consisted of a 7 spoke 1 hub telestroke system. It was designed to serve rural, remote, or neurologically underserved communities.

The original Arizona telestroke network recently celebrated its 10th year anniversary inspiring a look back at the original but unpublished data and a look forward at all the subsequent developments built on the network's foundational learning.

### Objective section

The STARR network registry consisted of a 7 spoke 1 hub telestroke system and its objective was to prospectively collect, record, and regularly analyze telestroke patient consultations and care data for the purposes of establishing quality measures, improvement, and benchmarking against other national and international telestroke programs.

## Methods

STARR, a prospective, single-arm, multicenter trial, was open to enrollment from October 24, 2008 to March 31, 2011 (29 months, total). Mayo Clinic Hospital, Phoenix, Arizona served as the hub primary stroke center and its vascular neurologists provided emergency telestroke consultations to seven participating rural, remote, or underserved spoke community hospitals in Arizona. Refer to Table 1 with spoke hospital characteristics. The nature of the relationships between STARR hub and spoke hospitals has already been described in two earlier publications.^4,5^

**Table 1.** Spoke Hospital Characteristics

Spoke hospital	Population of city (2010 census)	Urban area	Bed capacity of hospital	Trauma center level	Certified advanced life support hospital	Start date	Distance from hub (miles)	Distance from hub (kilometers)	Local neurologists	Annual stroke admissions (2012)	Heliport yes/no
Copper Queen Community Hospital	5575	No	25/(25^*^)	IV	No	January, 2009	236	380	0	0	Yes
Bisbee, AZ (Cochise County)^*^											
Verde Valley Medical Center	11,265	No	110	IV	Yes	September, 2009	97.6	157	3	94	Yes
Cottonwood, AZ (Yavapai County)											
Flagstaff Medical Center	65,870	Yes	267	I	Yes	July, 2010	139	224	5	230	Yes
Flagstaff, AZ (Coconino County)											
Kingman Regional Medical Center	28,068	No	235	IV	Yes	October, 2008	187	302	3	116	No
Kingman, AZ (Mohave County)											
La Paz Regional Hospital	3046	No	39/(25^*^)	IV	Yes	January, 2009	116	187	0	10	Yes
Parker, AZ (La Paz County)^*^											
Maricopa Integrated Health System	1,563,025	Yes	449	I	Yes	September, 2010	21.1	34	156	102	Yes
Phoenix, AZ (Maricopa County)											
Yuma Regional Medical Center	93,064	Yes	406	N/A	Yes	October, 2008	206	331	8	334	Yes
Yuma, AZ (Yuma County)											
	(1)	(9)	(2)	(7)	(7)	(3)	(4)	(4)	(5)	(7)	(8)

(1) Per City.Data.com.

(2) Per Hospital Website.

(3) Per Telestroke Newsletter 8/2011.

(4) Per MapQuest.

(5) In city per AZ Medical Board.

(6) From Mayo Telestroke Business Plan (Maricopa Integrated Health System not included).

(7) Per AZ Dept. Of Health Services—used Stroke discharge figures.

(8) FAA per City.Data.com.

(9) Per 2010 Census.

^*^ Critical Access Hospital with no more than 25 beds—per AZ Center for Rural Health.

Eligibility criteria for activation of a telestroke alert and study enrollment were: adult (age ≥18 years), suspicion of an acute stroke syndrome by emergency medical services (EMS) and emergency department (ED) triage, objective signs of focal neurological deficits on ED arrival, Cincinnati Prehospital Stroke Scale score of ≥1, and time window since onset or since last known to be at baseline state was 12 h. Exclusion criteria were: inability to obtain consent and incarceration.

The seven participating spoke hospitals included Copper Queen Community Hospital (Bisbee, AZ), Verde Valley Medical Center (Cottonwood, AZ), Flagstaff Medical Center (Flagstaff, AZ), Kingman Regional Medical Center (Kingman, AZ), La Paz Hospital (Parker, AZ), Maricopa Integrated Health System (Phoenix, AZ), and Yuma Regional Medical Center (Yuma, AZ). The distances between the hub and the closest and farthest spoke hospital were 21 and 236 miles, respectively (34 and 380 kilometers). The bed capacity of the spoke hospitals ranged between 25 and 449 (Refer to Table 1).

The hub telestroke consultation team included five American Board of Psychiatry and Neurology ABPN-certified vascular neurologists, one American College of Graduate Medical Education (ACGME) vascular neurology fellow, and one Neurovascular Education and Training in Stroke Management and Acute Reperfusion Therapies (NET-SMART) graduate vascular neurology nurse practitioner who were supervised by a vascular neurologist. The telestroke service operated 24/7/365.

Each spoke hospital's regional EMS providers, ED physicians, nurses, hospitalists, intensivists, radiologists/teleradiologists, radiology technologists, pharmacists, laboratory technologists, and information technologists received telemedicine orientation and training, in addition to acute stroke education. The hub personnel helped the spoke personnel to learn how to conduct the National Institutes of Health Stroke Scale (NIHSS) and to adopt a uniform stroke alert system, acute stroke care algorithm, and common paper or electronic acute stroke order sets, which adhered to the applicable American Heart Association/American Stroke Association (AHA/ASA) guidelines at the time of the study.^6^

Members of the Mayo Clinic hub telestroke team (including medical director, program manager, operations administrator, stroke nurse practitioner, clinical coordinator, emergency department (ED) nurse, Information Technology (IT), and clinical research coordinator) spent a minimum of 2 days at each spoke hospital campus before go-live and at least one day annually thereafter for ensuring telestroke skill maintenance and for providing acute stroke continuing medical education.

Every hub vascular neurologist was licensed in the state of Arizona and underwent credentialing, privileging, and requisite annual telemedicine practice peer reviews specified by each of the participating spoke hospitals. A standard telestroke service line contract agreement was signed by hub and spoke hospital officials. The state government sponsor, ADHS research grant covered the cost of acquisition, maintenance, and service of the selected telemedicine equipment as well as the professional telestroke services provided. During the study interval, spoke hospitals did not pay the hub hospital an annual subscription fee. Professional reimbursement for each telestroke consultation was not sought from government or nongovernment payers during the study period.

The STARR algorithm began with community emergency medical services (EMS) and/or spoke hospital ED personnel recognition of a possible acute stroke syndrome meeting STARR eligibility criteria (see above). Pre-hospital notification to the intended recipient hospital was triggered by EMS. The spoke ED activated a toll free telestroke hotline number, which resulted in a direct connection to either the hub on-call vascular neurologist's digital pager and/or cellular phone. No intermediate switchboard operator was positioned between the referring spoke hospital and the on-call vascular neurologist.

According to the STARR protocol algorithm, the hub vascular neurologist was obligated to respond by telephone within 10 min and by video telemedicine within 20 min of receipt of telestroke alert activation. In the event of no telephone response in 10 min, the spoke hospital re-activated the telestroke hotline and/or dialed hub hospital switchboard to be connected to the vascular neurologist on-call. These time intervals served as targets and were the subject of on-going quality assurance and quality improvement initiatives.

The STARR telestroke consultation overview, its features, suggested target time intervals, and an illustrative case has been previously published.^5^

The STARR trial protocol PR08-005156-09 was reviewed and approved by Mayo Clinic IRB, which served as the central IRB of record for all the participating hospitals. The STARR trial was registered at ClinicalTrials.gov.

### Technology methodology section

The equipment and software utilized in the STARR study could be grouped into three main categories: (1) telemedicine video cart systems, (2) picture archive and communications systems (PACS) software, and (3) call management solutions.

Telemedicine video cart systems used as communications endpoints at spoke hospitals included BF Technologies Telemedicine Carts (BF Technologies, Inc., San Diego, CA) and comprised Windows-based PC's running AccessVideo™ software to deliver secure Health Insurance Portability and Accountability Act (HIPAA) compliant two-way audio/high resolution video over standard Ethernet dedicated Internet services. In addition, GlobalMed Pole Series Mobile Video Carts (GlobalMedia Group, LLC, Scottsdale, AZ), which combined a Windows-based PC, integrated iRez i5770 pan/tilt/zoom camera, and EasyShare VC software, were used to provide secure HIPAA compliant two-way audio/video conferencing sessions at several spoke hospitals.

PACS software implemented for access to digital imaging and communications in medicine (DICOM) files included OnePacs, ResolutionMD^®^, and PacsOne (part of the turnkey BF Technologies Telemedicine Cart solution). The OnePacs (OnePacs, LLC, Wilmington, DE) system stored and transmitted DICOM data that could be accessed via a web-based study viewer or locally on server-based software installed at each spoke site. ResolutionMD (Calgary Scientific, Calgary, AB) enabled access to DICOM radiology images from web browsers and mobile devices by implementing proprietary U.S. Food and Drug Administration Class II accredited technology that safeguarded patient data behind the spoke hospital's firewall.

Finally, PacsOne, a DICOM 3.0 compliant PACS application, was implemented in conjunction with all BF Technologies Telemedicine Cart installations. Lastly, RingCentral (RingCentral, Inc., Belmont, CA), a cloud-based communications solution, was used to automatically direct incoming telestroke alert calls from spoke hospitals to the on-call vascular neurologist's digital pager and/or cellular phone based on pre-programmed daily schedules.

### Statistics section

Summary statistics were calculated for each variable.

## Results

**FIG. 1.**
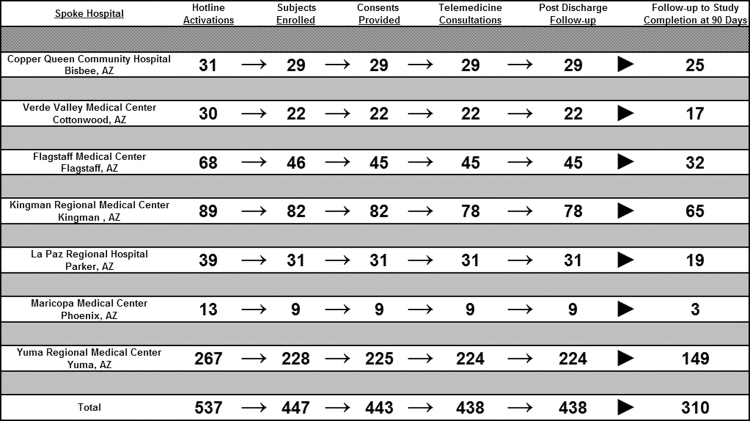
STARR trial subject enrollment to follow-up flow chart. STARR, Stroke Telemedicine for Arizona Rural Residents.

The telestroke hotline was activated 537 times by participating spoke hospitals during the study period. Figure 1 graphically displays the study flow: 537 telestroke hotline activations (telestroke alerts), 447 subjects who met eligibility criteria, and 443 subjects who provided consent for participation, to the 443 subjects who underwent telemedicine consultation and who had follow-up to hospital discharge, and finally to the 310 subjects for whom follow-up data were retrieved to study completion at 90 days.

Table 2 displays the number of subjects enrolled by each of the seven participating spoke hospital sites, ranging from 9 to 225 subjects and the total enrollment of 443 subjects.

**Table 2.** Stroke Telemedicine for Arizona Rural Residents Subject Enrollment by Spoke Hospital Site

	Total (***N*** = 443)
Site	
Yuma	225 (50.8%)
Kingman	82 (18.5%)
Parker	31 (7.0%)
Bisbee	29 (6.5%)
Cottonwood	22 (5.0%)
Flagstaff	45 (10.2%)
Maricopa	9 (2.0%)

For 68.2% (302/443) of subjects, the precise date and time of stroke symptom onset was discoverable and documented and for the remainder it was unknown. In 14.2% (63/443) of instances, the subject awoke from sleep with their neurological deficit. The mode of arrival at spoke hospital ED was ground ambulance in 65.7% (291/443), air ambulance in 0.5% (2/443), private automobile in 33.4% (148/443), in-patient in 0.2% (1/443), and not documented in 0.2% (1/443). The first documented blood pressure values were 151 mm Hg median systolic (Q1–136, Q3–170; range 96 to 240) and 87 mm Hg median diastolic (Q1–76, Q3–96; range 50 to 186).

Table 3 displays all the applicable acute stroke time intervals, from symptom onset (or time last known to be at baseline state) to 9-1-1 call and EMS transport times (when applicable), to spoke hospital arrival and triage, telestroke alert activation, consultation, diagnostic test availability and interpretation, preliminary diagnosis, thrombolysis decision making, thrombolysis administration, and telestroke consult completion.

**Table 3.** Stroke Telemedicine for Arizona Rural Residents Acute Stroke Time Intervals

	***N***	Mean	Standard deviation	Median	Q1, Q3
Symptom onset to ED arrival (min)	443	216.7	1333.5	66.0	40.0, 150.0
ED arrival to telestroke hotline page (min)	443	46.5	56.8	32.0	15.0, 57.0
Telestroke hotline page to neurologist returning call	443	1.3	2.5	1.0	0.0, 2.0
Neurologist returning call to consent signed (min)	443	14.2	168.6	4.0	0.0, 14.0
Consent signed to telemedicine consult commencing (min)	443	2.9	169.9	11.0	3.0, 20.0
Telemedicine consult commencing to alteplase eligibility decision	443	23.8	17.1	22.0	14.0, 31.0
Alteplase eligibility decision to alteplase administration (min)	100	23.3	13.3	24.0	15.0, 32.0
Alteplase eligibility decision to end of telemedicine consultation (min)	443	13.2	13.7	8.0	5.0, 17.0
Symptom onset to alteplase administration (min)	100	164.5	45.1	156.5	130.0, 196.0
ED arrival to alteplase administration (min)	100	98.4	29.4	95.5	81.0, 112.0
Symptom onset to EMS dispatch (min)	293	105.0	194.4	25.0	10.0, 99.0
EMS dispatch to EMS arrival on scene (min)	293	6.8	6.5	6.0	4.0, 8.0
EMS arrival on scene to EMS departure (min)	293	11.3	4.4	11.0	8.0, 14.0
EMS departure to ED arrival (min)	293	15.9	9.6	14.0	9.0, 21.0

ED, emergency department; EMS, emergency medical services.

Five vascular neurologists participated in the study, with a range of individual contributions. In descending order, vascular neurologists contributed 180, 102, 80, 72, and 9 of the 443 subjects' telestroke consultations during the study. For 40 out of 443 (9.0%) an ACGME vascular neurology fellow and in 23 out of 443 (5.2%) an NET SMART trained vascular neurology nurse practitioner contributed to the telestroke assessment under direct vascular neurology supervision.

Subject demographics include median age 69.1 years (Q1–56.5, Q3–78.7, range 21 to 97, ethnicity and race 423/443 [95.5%] White, 9/443 [2.0%] Black, 7/443 [1.6%] Native American Indian, 2/443 [0.5%] Pacific Islander, 1/443 [0.2%] Asian, and 1/443 [0.2%] Other/Not specified). Of all the subjects, 346/443 (78.5%) were non-Hispanic, 95/443 (21.5%) Hispanic, and 2/443 (0.5%) Not specified.

The baseline medical history and vascular risk factors of the participating subjects included coronary artery disease in 126 out of 443 (28.4%), diabetes mellitus in 118 out of 443 (26.6%), myocardial infarction in 81 out of 443 (18.3%), hypertension in 310 out of 443 (70.0%), congestive heart failure in 34 out of 443 (7.7%), stroke in 106 out of 443 (23.9%), peripheral arterial disease in 23 out of 443 (5.2%), hyperlipidemia in 211 out of 443 (47.6%), transient ischemic attack in 58 out of 443 (13.1%), atrial fibrillation in 69 out of 443 (15.6%), obstructive sleep apnea in 22 out of 443 (5.0%), family history of stroke in 88 out of 443 (19.9%), current smoking in 104 out of 443 (23.5%), smoking history in 207 out of 443 (46.8%), current alcohol consumption in 160 out of 443 (36.1%), and alcohol history in 195 out of 443 (44.0%).

The medication use of the participating subjects included anticoagulant in 30 out of 443 (6.8%), antiplatelet in 237 out of 443 (53.4%), lipid-lowering medication in 176 out of 443 (39.7%), anti-hypertensive medication in 272 out of 443 (61.4%), and hypoglycemic medication in 94 out of 443 (21.2%).

Urine pregnancy and drug toxicology screens were ordered by clinical investigator staff only when they were felt to be indicated. Urine pregnancy tests were ordered in 11 subjects, and all were negative. Urine drug toxicology screens were ordered in 76 out of 443 (17.2%) and of those ordered, 22 out of 76 (28.9%) were positive.

Subjects' pre-stroke Modified Rankin Scale score mean and standard deviation (SD) was 0.5 (1.0) with a range of 0 to 4, Modified Rankin Scale score at ED presentation mean and SD was 2.8 (1.5) with a range of 0 to 5, and NIHSS score mean (SD) at presentation was 8.1 (8.4) with a range of 0 to 40.

Non-contrast computed tomography (NCCT) head studies were acquired in all subjects and interpreted independently by a local spoke hospital radiologist and hub teleneurologist in 315 out of 443 (71.1%) and exclusively by the local spoke hospital radiologist in 128 out of 443 (28.9%). The overall quality of the scans was determined to be excellent in 347 out of 443 (78.3%), fair but interpretable in 17 out of 443 (3.8%), and quality not assessed in 79 out of 443 (17.8%). The NCCT was judged to be abnormal in 260 out of 443 (59.1%).

Radiologic evidence of an acute stroke was reported in 92 out of 443 (20.8%), chronic stroke in 199 out of 443 (44.9%), and signs of edema present in 14.6% (in those subjects with NCCT signs of edema present, sulcal effacement was identified in 54.1%, compression of ventricles in 40.5%, midline shift in 24.3%, and loss of gray-white differentiation in 67.6%), hyperdense artery signs in 17.8%, intracerebral hemorrhage in 8.1% [intracerebral hemorrhage (ICH) median volume 30.4 mL, Q1–11.7, Q3–89.3, range 1.9 to 340 mL], subarachnoid hemorrhage in 4.2%, acute subdural or epidural hematoma in 1.2%, intracranial neoplasm in 1.9%, cerebral atrophy in 29.2%, aneurysm clip in 0.4%, cyst in 0.4%, shunt in 1.2%, evidence of previous craniotomy in 2.3%, hydrocephalus in 0.8%, vertebral basilar dolichoectasia in 0.8%, parenchymal calcium deposition in 3.8%, and chronic small vessel ischemic changes in 28.8%.

Radiologic contraindications to intravenous thrombolysis were identified in 17.4% of NCCTs. Mean (SD) ASPECT score was 9.7 (0.9).

Table 4 displays the applicable exclusion criteria for alteplase in the study, and the proportion of subjects in the study identified as having one of the alteplase contraindications.

**Table 4.** Reasons Why IV Alteplase Was Withheld in Stroke Telemedicine for Arizona Rural Residents Trial

	***N* = 443** ***n* (%)**
<18 years of age	0 (0)
>80 years of age^*^	24 (5.4)
Uncertainty over the diagnosis and/or onset not clearly defined	164 (37.0)
No serious measurable deficit	178 (40.2)
Symptom onset >4.5 h	114 (25.7)
Minor and/or rapidly improving symptoms	201 (45.4)
NIHSS >25^*^	16 (3.6)
Evidence of ICH on pretreatment head CT scan	23 (5.2)
Presentation suggested SAH even if CT is normal	7 (1.6)
Female and known or suspected pregnancy	0 (0)
Platelet count <100,000	11 (2.5)
Elevated PTT	11 (2.5)
INR >1.7	12 (2.7)
Warfarin, regardless of INR^*^	25 (5.6)
Major surgery/serious trauma within 14 days	6 (1.4)
Recent intracranial or spinal surgery within 14 days	19 (4.3)
Recent serious head trauma within 3 months	11 (2.5)
History of GI or GU bleeding within 21 days	4 (0.9)
Arterial puncture at non-compressible site	0 (0)
Lumbar dural puncture within 7 days	0 (0)
Repeated SBP >185 or DBP >110 mm Hg despite antihypertensive treatment	32 (7.2)
Stroke within previous 3 months	12 (2.7)
History of both stroke and diabetes mellitus^*^	35 (7.9)
History of ICH	17 (3.8)
Serious medical illnesses likely to interfere with treatment	12 (2.7)
Life expectancy <1 year	5 (1.1)
Abnormal blood glucose accounting for deficit <50 mg/dL (or >400 mg/dL)	4 (0.9)
Presentation consistent with acute MI or pericarditis	5 (1.1)
Seizure at onset of stroke	21 (4.7)
Preexisting neurological/psychiatric disease confounding evaluation	18 (4.1)
Large area of obvious low-density lesions on CT head consistent with infarction	11 (2.5)
Left heart thrombus	1 (0.2)
Stroke team unable to determine eligibility	12 (2.7)
Patient or family refused thrombolysis	2 (0.5)
Delayed patient arrival	71 (16.0)
Failure to diagnose in eligible time window^*^	49 (11.1)
In-hospital time delay	21 (4.7)
No IV access	0 (0)
Other, unspecified	54 (12.2)

For some subjects, more than one exclusion criterion may have applied.

^*^ Applicable only to subjects within the extended 3 to 4.5 h time window.

CT, computed tomography; DBP, diastolic blood pressure; GI or GU, gastrointestinal or genito-urinary; ICH, intracerebral hemorrhage; INR, International Normalized Ratio; MI, myocardial infarction; NIHSS, National Institutes of Health Stroke Scale; PTT, partial thromboplastin time; SAH, subrachnoid hemorrhage; SBP, systolic blood pressure.

In 291 out of 443 subjects (65.7%), the onset of the stroke was witnessed by a family member, friend, companion, or bystander who accompanied the subject to the ED. During the telestroke consultations, the neurologists obtained information regarding the subject's history from the patient directly (323/443 (72.9%)), a family member (303/443 (68.4%)), a companion (32/443 (7.2%)), an ED nurse (282/443 (63.7%)), an ED physician (328/443 (74.0%)), an ED resident (37/443 (8.4%)), and/or other (14/443 (3.2%)).

Neurologists received telepresenter bedside assistance with their telemedicine examination of the subject in the ED by the ED nurse (376/443 (84.9%)), ED physician (89/443 (20.1%)), ED resident (27/443 (6.1%)), mid-level practitioner (1/443 (0.2%)), family member or companion (66/443 (14.9%)), and/or other (20/443 (4.5%)).

### Technical observations

The telestroke consultations began in 437 out of 443 (98.6%) subjects and they were successfully completed in 430 out of 443 (97.1%) subjects. The 7 telestroke consultations that started but could not be completed each failed due to an Internet connectivity issue, which could not be immediately rectified.

In 254 out of 443 (57.3%) of the subjects' telestroke consultations, a technical observation arose. Of all the subjects' consultations with technical observations, 44.1% included an audio problem, 48.8% included a video problem, 51.6% included a radiology problem, 20.5% included an Internet connection problem, and 11.8% included a problem not otherwise classified. Of the 254 subjects' consultations with technical observations, 181 out of 254 (71.3%) had no effect on clinical diagnosis and decision making, 69 out of 254 (27.2%) delayed clinical diagnosis and decision making, and only 4 out of 254 (1.6%) prevented a clinical diagnosis and/or decision making from occurring.

### Telemedicine equipment

The technology deployed during the consultations included the telephone in 435 out of 443 (98.2%), BF Technologies (BFT) audio video in 297 out of 443 (67.0%), BFT DICOM in 297 out of 443 (28.0%), Global Med audio video in 103 out of 443 (23.3%), iPhone Calgary Scientific in 98 out of 443 (22.1%), OnePACS teleradiology in 84 out of 443 (19.0%), Computer PACSone in 82 out of 443 (18.5%), iPhone PACSone in 17 out of 443 (3.8%), and other in 4 out of 443 (0.9%). The most frequently preferred sequence of technology deployment during telestroke consultations was first telephone communication, second audio video telemedicine connection, and third teleradiology access.

### ED admitting diagnoses

After the telestroke consultations, the ED admitting diagnoses were ischemic stroke with administration of intravenous (IV) alteplase in 100 out of 443 (22.6%), ischemic stroke without IV alteplase administration in 170 out of 443 (38.4%), transient ischemic attack in 30 out of 443 (6.8%), intracerebral hemorrhage in 21 out of 443 (4.7%), subarachnoid hemorrhage in 2 out of 443 (0.5%), non-cerebrovascular related in 59 out of 443 (13.3%), and unknown in 61 out of 443 (13.7%).

### Follow-up imaging

The CTA head and neck studies were conducted in 99 out of 443 (22.3%). In 36 out of 99 (36.4%), an arterial occlusion was observed; in ICA, 22 out of 99 (22.2%), MCA-M1 11 out of 99 (11.1%), MCA-M2 6 out of 99 (6.1%), ACA 3 out of 99 (3.0%), Basilar 1 out of 99 (1.0%), and PCA 0 out of 99 (0%). The magnetic resonance angiography head and neck studies were conducted in 206 out of 443 (46.5%) and 51 out of 443 (11.5%), respectively. Carotid ultrasound studies were conducted in 235 out of 443 (53.0%), echocardiogram trans-thoracic studies in 197 out of 443 (44.5%), and trans-esophageal in 52 out of 443 (11.7%).

Magnetic resonance imaging (MRI) brain studies were conducted in 307 out of 443 (69.3%) and in 108 out of 307 (58.6%) a clinically relevant acute ischemic stroke was identified. The NCCT studies were conducted within 48 h post-stroke in 134 out of 443 (30.2%), and a clinically relevant acute ischemic stroke was identified in 72 out of 134 (53.7%). Mean (SD) ASPECT score was 7.9 (3.0) with range 0–10. Hyperdense MCA sign was identified in 27 out of 134 (20.1%) of the NCCT studies.

### Thrombolysis complications

The IV alteplase was administered to 100 out of 443 (22.6%) of the subjects in the study. Post-thrombolysis, any intracerebral hemorrhage was detected in 12 out of 100 (12.0%), symptomatic ICH in 5 out of 100 (5.0%), and asymptomatic ICH in 7 out of 100 (7.0%). ECASS HI-1 was detected in 2 out of 12 (16.7%), HI-2 in 1 out of 12 (8.3%), PH-1 in 3 out of 12 (25.0%), and PH-2 in 5 out of 12 (41.7%). The outcomes post-SICH were 4 out of 12 (33.3%) complete recovery, 5 out of 12 (41.6%) recovery with sequelae, and 3 out of 12 (25.0%) fatal. Major systemic bleeding occurred in 2 out of 100 (2.0%) of subjects receiving IV alteplase, and hemilingual angioedema occurred in 1 out of 100 (1.0%).

Follow-up brain imaging studies confirmed the presence of an acute ischemic stroke in 233 out of 443 (52.6%) of subjects overall.

### Etiologic classification of ischemic stroke

Etiologies of the ischemic stroke were classified by TOAST criteria as follows: Large Artery Atherosclerosis 11.8%, Cardioembolic 48.1%, Small Vessel (Lacunar) 17.5%, Stroke of Other Determined Etiology 3.0%, and Stroke of Undetermined Etiology 19.5%.

### Interventional management

Interventions of any kind occurred in 36 out of 443 (8.1%) of the subjects. The interventions were (arranged from most to least frequent) other 23, craniotomy for ICH evacuation 4, Inferior Vena Cava filter placement 4, carotid angioplasty and stenting 3, decompressive hemicraniectomy 3, cardiac catheterization 3, carotid endarterectomy 2, endovascular thrombectomy 2, and intracranial aneurysm coiling 1 (note some subjects had ≥1 intervention).

Table 5 lists all the subjects' final hospital discharge diagnoses. Approximately half of the subjects were diagnosed with an ischemic stroke, and approximately three quarters of the subjects were diagnosed with a cerebrovascular disease of some variety (ischemic or hemorrhagic). The list of non-cerebrovascular diseases mimicking stroke is provided in Table 5.

**Table 5.** List of Stroke Telemedicine for Arizona Rural Resident Subjects' Final Hospital Discharge Diagnoses

	***N* = 443 *n* (%)**
Ischemic stroke	234 (52.8)
TIA	65 (14.7)
ICH	23 (5.2)
SAH	2 (0.5)
Encephalopathy	6 (1.4)
Seizure	21 (4.7)
Syncope/presyncope	7 (1.6)
Migraine	10 (2.3)
Functional	15 (3.4)
Dementia	5 (1.1)
Vestibular neurontitis/labyrinthitis/BPPV	2 (0.5)
Meningitis/encephalitis	1 (0.2)
Brain neoplasm	4 (0.9)
Hypoglycemia	1 (0.2)
Unknown diagnosis	11 (2.5)
Transient global amnesia	2 (0.5)
Other/not classifiable	34 (7.7)

BPPV, benign paroxysmal positional vertigo; TIA, transient ischemic attack.

Subjects' disposition status after ED assessment was 312 out of 443 (70.4%) admitted to Spoke Facility (Originating Site) and 131 out of 443 (29.6%) transferred from Spoke Facility (Originating Site). Of those subjects transferred from Spoke Facility to another facility, 29% were transferred to Mayo Clinic Hospital Stroke Center in Phoenix and 71% were transferred to another regional stroke center. The units to which the subjects were admitted were 103 out of 443 (23.3%) intensive care unit (ICU), 199 out of 443 (44.9%) Medical-Surgical Telemetry Unit, 41 out of 443 (9.3%) Discharged home, 4 out of 443 (0.9%) deceased, and 96 out of 443 (21.7%) Other/Unclassified.

The 90-day follow-up was conducted by neurology nurse research coordinators directly with subjects by telephone whenever feasible, by delegated caregivers or family members whenever necessary, and supplemented by paper and electronic medical records from health care providers. A 90-day follow-up was successfully conducted for 310 out of 443 (70.0%) of subjects. The subjects' 90 days Modified Rankin Score and Barthel Index Score are presented in Table 6.

**Table 6.** The Subjects' 90 Days Modified Rankin Score and Barthel Index Score

Modified Rankin Score	
N	310
0-No symptoms at all	80 (25.7%)
1-No significant disability	95 (30.5%)
2-Slight disability	32 (10.3%)
3-Moderate disability	43 (13.8%)
4-Moderately severe disability	27 (8.7%)
5-Severe disability	6 (1.9%)
6-Death	28 (9.0%)

Barthel Index-Total Score	
N	283
Mean (SD)	88.9 (22.3)
Median	100.0
Q1, Q3	90.0, 100.0
Range	(0.0-100.0)

Place of residence, for those subjects with 90 days follow-up, was distributed as follows: House/Apartment (independent) 64.0%, House/Apartment (dependent) 28.6%, Nursing Home/Extended Care Facility 2.8%, Assisted Living Facility 1.1%, Rehabilitation Facility 3.2%, and Other not classified 0.3%. The living status in those subjects with 90-day follow-up data was 91.6% alive, 8.4% deceased.

## Discussion

As specified at the beginning of this article, the original Arizona telestroke network celebrated its 10th year anniversary in 2021, inspiring inspection and publication of the original trial data, assessment of how successfully the original objectives were met, recognizing all the subsequent developments built on the network's foundational learning, and taking a glance forward to the future.

The STARR trial objective was to assess the feasibility of prospective collection, recording, and regularly analyzing telestroke patient consultations and care data for the purposes of establishing quality measures, improvement, and benchmarking against other national and international telestroke programs.

The success of STARR included the recruitment of multiple disparate and geographically separated rural and remote hospitals to collaborate in a novel state-wide government funded digital health research study, development of telestroke service contracts, utilization of a centralized IRB for telemedicine research, adoption of a hybrid of in-person and virtual research trial teaching, learning, and ongoing communication as well as trial coordination, utilization of an evidence-based pragmatic telestroke clinical algorithm across the network, documentation of standardized data entry fields for telestroke clinical care and research, incorporation of stroke specialty trained fellows and nurse practitioners into telestroke practice and research, deployment and evaluation of a multitude of telemedicine technologies in a single trial, and acquisition of complete data through to the conclusion of subjects' hospitalization for stroke despite operating within different electronic medical records.^7,8^

A weakness in STARR is the 133 out of 443 (30%) of subjects for whom 90-day follow-up data could not be determined. This was anticipated to be a potential issue given that subjects were generally residing in remote and rural communities in the state: Some were non-residents, some were vacationers, some were homeless, and some reported no permanent address.

The STARR network was very much comparable to the Finnish telestroke network (FIN) for benchmarking, as each was at the 2 years mark in 2011.^9^ Sixty one (FIN) and 100 (STARR) acute ischemic stroke subjects, respectively, had already received IV alteplase in the first 2 years of the programs. Mean age was comparable: 70 (FIN) and 69 (STARR). Fifty-one percent (FIN) and 50% (STARR) female, median and range of subjects' NIHSS were 10 and 3–26 (FIN) and 8 and 0–40 (STARR).

Telestroke consultation duration was nearly identical, 25 min (FIN) and 24 min (STARR). Mean onset to treatment time and SD was 130 and 45 min (FIN) and 165 and 45 min (STARR). The proportion of telestroke patients who received IV alteplase was higher (61/106 (57.5%)) in Finland and relatively lower (100/443 (22.6%)) in STARR. However, a higher percentage of patients in FIN versus STARR had acute ischemic stroke versus non-cerebrovascular diagnoses. If one looked at the proportion of STARR patients with ischemic stroke who received IV alteplase, the result was 100 out of 234 (42.7%), which was more similar to FIN experience.

Symptomatic intracranial hemorrhage post-thrombolysis was similar between these networks (6.7% (FIN) and 5.0% (STARR)). In hospital or end-of-stay mortality was 9.8% (FIN) and 8.4% (STARR). Forty-nine percent (FIN) and 56% (STARR) of telestroke patients had a favorable outcome.

Reasons to withhold IV alteplase were similarly reported across the two trials: mild or quickly resolving symptoms 49% (FIN), 45% (STARR), stroke <3 months 4% (FIN), 3% (STARR), dependent for activities of daily living (mRS >2) 4% (FIN & STARR), NIHSS >25 in 4% (FIN & STARR), intracranial hemorrhage on computed tomography (CT) 4% (FIN), 5% (STARR), INR >1.7 in 2% (FIN), 3% (STARR), history of diabetes and previous stroke 2% (FIN), 8% (STARR), uncontrolled hypertension 4% (FIN), and 7% (STARR).

However, the trials differed in their respective reporting of etiologies other than stroke, resulting in withholding of IV alteplase 15% (FIN) and 37% (STARR) and symptom onset >3 h in 9% (FIN) and 26% (STARR), which may reflect trial differences in telestroke alert activation criteria and any pre-consultation screening protocols.

The NCCT head studies were acquired in all subjects and interpreted independently by a local spoke hospital radiologist and hub teleneurologist in 315 out of 443 (71.1%) and exclusively by the local spoke hospital radiologist in 128 out of 443 (28.9%).^10,11^

When STARR is compared against a nationally agreed-on minimum data set of variables designed to evaluate new and existing telestroke networks and services, STARR achieved 14 out of 16 (88%) Suggestions for Measuring Telestroke Processes (lacking tracking transfers between facilities to understand the flow of patients, cost structure, and eventual outcomes, times of transfer, and arrival at destination facility), 23 out of 28 (82%) Suggestions for Measuring Telestroke Outcomes (lacking ambulatory status, percent of all patients seen in the ED with the initial diagnosis of stroke and, when available, the percent of all patients discharged with a stroke diagnosis, percent of stroke patients arriving in the ED within the 3- and 4.5-h time windows since last known well, and percent of all stroke patients within these windows in whom documentation does not include a reason for not treating with intravenous alteplase).

STARR achieved 2 out of 6 (33%) Suggestions for Measuring Patient and Provider Satisfaction (STARR included surveys of technology, however it was lacking a satisfaction and experience monitoring program including satisfaction with the provider and staff, interactions, as well as the overall experience), and 11 out of 12 (92%) Suggestions for Monitoring Telestroke Technology Quality (lacking recording of any telestroke interactions in which a violation of security or protected health information policies was suspected as a result of technical problems to be recorded, investigated, and corrected).^12^

When STARR is compared against an internationally agreed-on minimum data set of variables designed to evaluate new and existing telestroke networks and services, STARR achieved 12 out of 12 (100%) Details about Telestroke Program/Network, 10 out of 10 (100%) Details about Initiating Hospital, 17 out of 17 (100%) Telestroke Consultation, 5 out of 7 (71%) Patient Characteristics (missing living arrangements before hospitalization and details regarding pre-notification), 5 out of 5 (100%) Presentation to hospital, 10 out of 10 (100%) General clinical care within first 24 h, 10 out of 10 (100%) Thrombolysis treatment, 13 out of 13 (100%) Endovascular treatment, 8 out of 8 (100%) Neurosurgery treatment, 7 out of 7 (100%) Processes of care beyond the first 24 h, 5 out of 5 (100%) Discharge information, and 6 out of 6 (100%) Post-discharge and follow-up.^13^

Based on the preliminary learning from the STARR trial and contributions from other national and international pioneers in telestroke, there have been many advancements and developments in Mayo Clinic telestroke and acute care telemedicine in general. Regionally focused telestroke networks at Mayo Clinic have converged into one enterprise-wide national telestroke network.^14,15^ Simultaneous use of multiple telemedicine vendor technologies has given way to the selection of a single highest performing vendor.^14^

Small teams of telestroke doctors stretched to their limits have ultimately collaborated in a single large deep pool of telestroke doctors, each suitably trained, licensed, and credentialed in multiple states and health systems.^16^ Disparate and variable oversight, management, operations, and administration have yielded to a centralized medical and administrative leadership structure and commonly used evidence-based management guidelines.^17^ Sporadic and episodic data collection has given way to continuous quality assurance and quality improvement according to national and international criteria.^12,13^

Telemedicine training programs for students, residents, fellows, and advanced practice providers are routinely available.^18,19^ Data from original telestroke networks have made health economic analyses possible.^20–23^ Prehospital telemedicine enabled ambulances and mobile stroke units complement the work of hospital-based telestroke networks.^24^ Since the learnings of 500 telestroke subjects in STARR, more than 32,000 telestroke patients have been assessed and treated at Mayo Clinic, enterprise wide.

Telestroke has helped spawn acute care telemedicine in various other acute care clinical domains, including hospital neurology, neurosurgery, neurocritical care, sports concussion, emergency psychiatry, emergency medicine, pediatrics, neonatology, obstetrics, and gynecology to name a few.^25–35^ Other multicenter national and international acute care telemedicine research consortiums have developed and are conducting feasibility and efficacy trials.^32^ Telestroke has matured beyond its original focus in the ED and now exists across the continuum of care.^36,37^ The telemedicine network's existence, maturity, and stability helped greatly during the coronavirus disease 2019 (COVID-19) pandemic and eased the stress endured by our health care system.^38–40^

## Conclusion

The emergence, success, and early learnings of a state-wide telestroke research network in a health care institution helped inform the development of larger multistate and national telemedicine networks as well as the extension of the acute care telemedicine model into other time-sensitive clinical care domains.

## Authorship Confirmation

All authors meet authorship criteria, have approved this article, and can take responsibility for all or part of the data.

## Abbreviations Used

ACGME: American College of Graduate Medical Education
ADHS: Arizona Department of Health Services
AHA/ASA: American Heart Association/American Stroke Association
BFT: BF Technologies
BPPV: benign paroxysmal positional vertigo
COVID-19: coronavirus disease 2019
CT: computed tomography
DBP: diastolic blood pressure
DICOM: digital imaging and communications in medicine
ED: emergency department
EMS: emergency medical services
FIN: Finnish telestroke network
GI or GU: gastrointestinal or genito-urinary
HIPAA: Health Insurance Portability and Accountability Act
ICH: intracerebral hemorrhage
ICU: intensive care unit
INR: International Normalized Ratio
IT: Information technology
IV: intravenous
MI: myocardial infarction
MRI: magnetic resonance imaging
NCCT: non contrast computed tomography
NET-SMART: Neurovascular Education and Training in Stroke Management and Acute Reperfusion Therapies
NIHSS: National Institutes of Health Stroke Scale
PACS: picture archive and communications systems
PTT: partial thromboplastin time
SAH: subrachnoid hemorrhage
SBP: systolic blood pressure
SD: standard deviation
STARR: Stroke Telemedicine for Arizona Rural Residents
STRokE DOC: Stroke Team Remote Evaluation Using a Digital Observation Camera
TIA: transient ischemic attack
